# Editorial: The use of repetitive peripheral magnetic stimulation (rPMS) in neurological disorders and neurorehabilitation

**DOI:** 10.3389/fneur.2023.1324882

**Published:** 2023-11-20

**Authors:** Volker Zschorlich, Tomofumi Yamaguchi, Cyril Schneider

**Affiliations:** ^1^Department of Movement Science, Faculty of Philosophy, Institute of Sport Science, University of Rostock, Rostock, Germany; ^2^Faculty of Human and Social Sciences, Institute of Sport Science, University of Oldenburg, Oldenburg, Lower Saxony, Germany; ^3^Department of Physical Therapy, Faculty of Health Science, Juntendo University, Tokyo, Japan; ^4^School of Rehabilitation Science, Faculty of Medicine, Université Laval, Québec, QC, Canada

**Keywords:** migraine, paresis, stroke, muscle spasticity, cerebral palsy, neurological disorder, tinnitus, pain

We are delighted to present a Research Topic of Frontiers in Neurology (basic neuroscience, clinical research) focused on the use of repetitive peripheral magnetic stimulation (rPMS) in neurorehabilitation. rPMS is a non-invasive electromagnetic transduction for stimulating nerves, muscles, spinal roots, or even the autonomic nervous system.

A crucial paradigm shift in healthcare has been brought about by non-invasive technologies that now enable non-invasive improvements of therapeutic interventions. This Research Topic aims to bring together research articles that explored various aspects of one non-invasive technology (rPMS), thus illuminating its use and promising results in different physiopathological conditions. rPMS, as other technological innovations used in neurorehabilitation, has evolved significantly in recent years and it became less expensive a technology than in its first years of testing. This may have contributed to its internationally enlarged popularity, given the increasing number of rPMS publications (see Figure 1) for diagnosis and treatment in the fields of neuroscience and neurorehabilitation.

**Figure 1:**
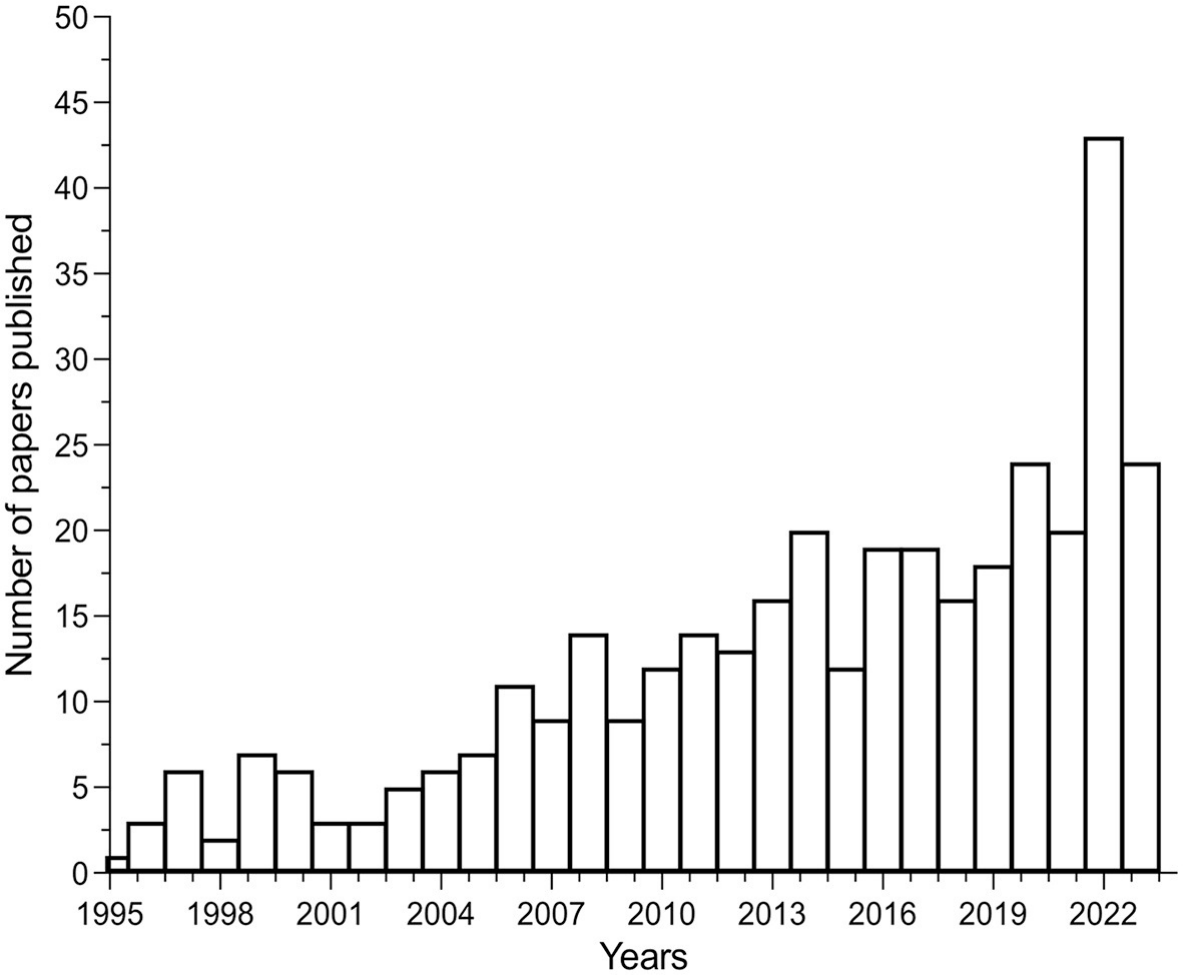
Increasing number of scientific papers over the last decades in the field of repetitive peripheral magnetic stimulation in neurorehabilitation.

Kolin et al. (1) originally showed that the application of magnetic stimulation over the gastrocnemius of a frog (nerve-muscle preparation) could induce muscle contraction. Research on the development of commercially available magnetic nerve and brain stimulators (2), the advent of repetitive magnetic stimulation (capable of generating multiple pulses per second) in the mid-1990s and the dire advantages of the magnetic neural stimulation over the electrical, all contributed to the increase of studies using rPMS, especially in the two last decades. E.g., rPMS presents with little decay of electrical current against the distance to the target tissue and negligible recruitment of skin nociceptors, thus activating deep-lying nerve branches or muscle fibers (3) at lower intensities than with electrical stimulation. Thus, rPMS is painless over most parts of the body and in most people (healthy or neurological population).

rPMS influences the excitability of the responsive structures beneath the coil, resulting in changes of neuronal function as reported at the cortical level as an influence of neuroplasticity (4, 5) that can in turn ease responsiveness of a patient to subsequent physical therapy (6). rPMS is simple to deploy in clinical settings and it can mimic the phenomenon of muscle contraction/relaxation giving rise to movement at far higher frequencies than possible by a therapist driving the movement.

The purpose of the present Research Topic, “*The use of repetitive peripheral magnetic stimulation (rPMS) in neurological disorders and neurorehabilitation*,” is to open the door for the use of rPMS as a tool alone or an adjuvant to be involved in the treatment of motor disorders in humans, as well as to lessen pain and other disorders in neurorehabilitation.

(1) The first paper by Schneider et al. “*Checklist on the Quality of the Repetitive Peripheral Magnetic Stimulation (rPMS) Methods in Research: An International Delphi Study*” is about a consensus statement for the correct and comprehensible description of stimulation protocols when using the bottom-up approach of rPMS. The consensus paper asked for important and significant information for replicability and understanding of the effects of the stimulation. The article provides an overview of the essential parameters and methodological steps that should be mentioned in any paper treating of rPMS and its clinical applications.

(2) A second paper from Börner et al. “*Response Predictors of Repetitive Neuromuscular Magnetic Stimulation in the Preventive Treatment of Episodic Migraine*” showed that the repetitive neuromuscular magnetic stimulation (rNMS) of the trapezius muscles could decrease headache and that the responsiveness was associated with the baseline characteristics of headache and the level of hyperalgesia of the neck muscles among patients. Such predictors are of utmost importance for the early identification of people with headache who would benefit from rNMS, thus, to ease the provision of an efficient tailored multimodal care planning.

(3) The third paper from Pan et al. “*Effects of repetitive peripheral magnetic stimulation on spasticity evaluated with modified Ashworth scale/Ashworth scale in patients with spastic paralysis: A systematic review and meta-analysis*” explored the impact of rPMS on motor function and everyday activities in persons with spastic paresis. Spasticity is a common motor disorder that is frequently caused by upper motor neuron lesion. This meta-analysis proposed that rPMS improved motor function and daily life activities among individuals with spastic paresis, and particularly enhanced the passive muscle characteristics examined by the modified Ashworth Scale.

(4) The last paper of this Research Topic by Grosse et al. “*Addressing gross motor function by functional repetitive neuromuscular magnetic stimulation targeting to the gluteal muscles in children with bilateral spastic cerebral palsy: benefits of functional repetitive neuromuscular magnetic stimulation targeting the gluteal muscles*” reported that the combination of repetitive neuromuscular magnetic stimulation (rNMS) of the gluteal muscles with a personalized physical training improved stability during standing and walking in children with cerebral palsy (CP). The authors suggested that rNMS represented a safe, well-feasible and clinically efficient approach able of increasing muscle weakness and enhancing selective motor control in spastic children with CP.

This Research Topic outlined that the gold-standard use of rPMS alone or as an adjuvant can improve the neurological condition in adults or children. This adds evidence of after-effects to rPMS literature in physiopathology of sensorimotor disorders or pain, and even in healthy people where rPMS-induced lowering of skeletal muscle reflex activity has further encouraged to develop clinical trials aiming at improving the condition of any people with hypertonia or spasticity. Overall, this Research Topic aligns with the literature to underline that the exploration of rPMS after-effects are worth be pursued with a view to improving the application procedures and going beyond the functional plateau already reached in neurorehabilitation.

Despite a growing evidence of rPMS beneficial effects in rehabilitation, some questions remained to be resolved in the future, e.g., the prediction of responsiveness (responders vs. non-responders), the application in other health conditions, the acceptability of implementation in clinical settings. It is therefore direly needed that researchers and practitioners carefully examine which aspects of rPMS-based rehabilitation improve its therapeutic efficacy, how to tailor rPMS as an adjuvant, and which neurophysiological mechanisms underpin the clinical changes.

## Author contributions

VZ: Conceptualization, Writing – original draft, Writing – review & editing. TY: Conceptualization, Writing – review & editing. CS: Conceptualization, Supervision, Visualization, Writing – review & editing.
